# Women had favourable reverse left ventricle remodelling after TAVR

**DOI:** 10.1111/eci.13183

**Published:** 2020-01-09

**Authors:** Su‐Chan Chen, Hsin‐Bang Leu, Hsiao‐Huang Chang, I‐Ming Chen, Po‐Lin Chen, Su‐Man Lin, Ying‐Hwa Chen

**Affiliations:** ^1^ Department of Medicine School of Medicine National Yang‐Ming University Taipei Taiwan; ^2^ Division of Cardiology Department of Medicine Taipei Veterans General Hospital Taipei Taiwan; ^3^ Healthcare and Management Center Taipei Veterans General Hospital Taipei Taiwan; ^4^ Division of Cardiovascular Surgery Department of Surgery Taipei Veterans General Hospital Taipei Taiwan; ^5^ School of Medicine Taipei Medical University Taipei Taiwan; ^6^ Division of Anesthesia Taipei Veterans General Hospital Taipei Taiwan

**Keywords:** aortic stenosis, LV remodelling, transcatheter aortic valve replacement

## Abstract

**Background:**

Being woman is associated with higher survival rates after transcatheter aortic valve replacement (TAVR) despite the increase in periprocedural complications. The left ventricle (LV) remodelling process that follows TAVR is considered to play an important role. We aim to investigate whether gender difference affects the process of LV remodelling after TAVR.

**Materials and Methods:**

A total of 100 patients (50 men and 50 women) after TAVR were enrolled. Echocardiography was performed at baseline before the TAVR procedure and repeated upon discharge, and at three, nine and 12 months post‐TAVR.

**Results:**

Women exhibited an early regression of LV mass and the LV mass index (LVMi) decreased 12.0% from 148.3 ± 48.0 to 130.5 ± 43.7 g/m2 at just a median of 17 days after the procedure (P < .001). Almost one‐half of the LVMi regression occurred by 17 days post‐TAVR and the LVMi regressed 22.0% by 12 months post‐TAVR. In contrast, the regression of LVMi in men seemed to be more gradual and the significant regression of LVMi from baseline began to be observed since three months later after TAVR. The LVMi reduction at nine months was 11.5% and achieved 15.4% over one year. Multivariable logistic regression analysis showed only the female sex, better LVEF and greater baseline LVMi were independently associated with greater LVMi regression after TAVR, indicating female gender is an independent predictor for favourable LV remodelling after TAVR.

**Conclusion:**

In conclusion, female patients with AS had favourable reverse remodelling with greater and earlier LV mass regression post‐TAVR compared with the male patients.

## 1 INTRODUCTION

With a reported prevalence of 12.4% among elderly patients, aortic stenosis (AS) is the most common form of valvular heart disease in the ageing population.^1^ Without valve replacement, the prognosis for hemodynamically severe and symptomatic AS is poor. Transcatheter aortic valve replacement (TAVR) has become the gold standard of care for patients with hemodynamically severe AS who are symptomatic but deemed to be too high‐risk for surgery. Recent reports suggest that sex‐based differences exist in terms of post‐TAVR clinical outcome. Being woman is associated with better mid‐ and long‐term survival following TAVR despite increased periprocedural complications, particularly more vascular complications (6%‐20% vs 2%‐14%) and higher bleeding rates (10%‐44% vs 8%‐25%) at 30 days post‐TAVI.^2^, ^3^, ^4^, ^5^, ^6^

Why female patients experience better outcomes remains poorly understood. The process of LV remodelling post‐TAVI likely contributes to the sex‐based differences in outcome after TAVI. The pressure overload in the LV caused by AS induces cardiac muscle hypertrophy and interstitial collagen deposition, leading to structural changes in the LV (ie LV remodelling). Sex appears to exert a strong influence on LV remodelling in surgical aortic valve replacement.^7^, ^8^, ^9^, ^10^ Women with severe AS typically manifest more concentric LV geometry, less myocardial fibrosis and better systolic function compared with their male counterparts. Surgical studies of patients with AS undergoing AVR have found less fibrosis on the myocardium biopsies from women, and the regression of the myocardial hypertrophy also occurs more rapidly in female patients.^11^ The goal of the present study was to investigate the timeline of LV remodelling in both sexes towards establishing whether women have more favourable outcomes relative to men and identifying independent predictors for early LV mass regression following TAVR.

## 2 METHODS

### 2.1 Patients

From May 2010 to March 2016, a total of 130 consecutive patients with severe, symptomatic AS (valve area ≤ 1.0 cm^2^) were evaluated. All patients had New York Heart Association (NYHA) symptoms exceeding class II and were slated to undergo TAVR. These patients were selected for TAVR after a heart team discussion deemed them either unsuitable or too high‐risk for surgical aortic valve replacement. Their individual operative risk was calculated using the logistic European System for Cardiac Operative Risk Evaluation (EuroSCORE) system. Patient selection was based on the approved indication for TAVR in Taiwan using either of the following criteria: (a) patients considered to be at high surgical risk with logistic EuroSCOREs ≥ 20%; (b) patients aged over 80 years old; (c) a history or the presence of previous cardiac surgery, porcelain aorta, thoracic burning sequelae contraindicating open heart surgery, mediastinum radiotherapy, severe connective tissue disease contraindicative to surgery, cirrhosis of the liver (child class A or B) or severe pulmonary insufficiency with forced expiratory volume in one second (FEV1) under one litre. The selection of patients for whom TAVR was determined to be the most suitable treatment approach required the clinical consensus of a multidisciplinary team comprised of cardiac surgeons, interventional cardiologists, anaesthetists and imaging specialists. The main exclusion criteria were a native aortic valve annulus of less than 18 mm or more than 29 mm, acute myocardial infarction for under 14 days, a left ventricular ejection fraction below 20%, active infection, hemodynamic instability or a life expectancy under 12 months. This study was approved by the institutional research board in Taipei Veterans General Hospital and followed STROBE statement and the broader EQUATOR guidelines.^12^

### 2.2 TAVR procedure

All TAVR procedures were performed in a specially‐equipped hybrid operating suite. At the beginning of this study, the TAVR procedures were performed under general anaesthesia. Since December 2013, local anaesthesia with conscious sedation has been used exclusively for transfemoral TAVR at our institution. The standard approach was through the transfemoral route, when feasible. For patients without the adequate anatomy required to allow for safe transfemoral access, alternative access routes were employed. Adjunct pharmacologic therapy included heparin treatment during the procedure and aspirin (100 mg/d) indefinitely and clopidogrel (75 mg/d) for three to six months following the procedure.

### 2.3 Echocardiography

Echocardiography was performed using a GE Vivid E9 system (GE Healthcare) with a 4‐MHz transducer. An independent core laboratory analysed all of the patient echocardiograms. The mass of the LV was calculated using the formula put forth by the American Society of Echocardiography and indexed to body surface area (termed LV mass index).^13^ The presence and severity of post‐procedural aortic regurgitation were determined according to the Valve Academic Research Consortium‐2 (VARC‐2) criteria.^14^ As described in their protocol, echocardiograms were obtained at baseline (within 45 days of the TAVR) and post‐TAVR at discharge (or 7 days), three months, nine months and one year.^13^

### 2.4 Clinical endpoints

The 30‐day combined safety endpoint is defined by the VARC‐2 [13] as a composite of all‐causes of mortality, major stroke, life‐threatening or disabling bleeding, acute stage 2 or 3 kidney injury including renal replacement therapy, major vascular complications and repeat procedure for valve‐related dysfunction. Stroke was defined as a focal neurologic deficit lasting over 24 hours or a focal neurologic deficit lasting under 24 hours with imaging findings of acute infarction or haemorrhage. The VARC‐2 proposed using the AKIN system for reporting acute kidney injury (AKI).

### 2.5 Statistical analysis

Data were expressed as the mean ± standard deviation for the numeric variables and as a per cent for the categorical variables. Continuous variable comparisons between groups were achieved using a one‐way analysis of variance (ANOVA). Subgroup comparisons among the categorical variables were assessed using the Chi‐square and Fisher's exact tests. Serial changes in LV mass index (LVMi) post‐TAVR were analysed using a paired *t* test, which enabled evaluation of the differences at each LVMi follow‐up. A logistic regression analysis was performed to investigate the independent factors potentially contributing to LV remodelling after the TAVR procedure.

## 3 RESULTS

**Figure 1:**
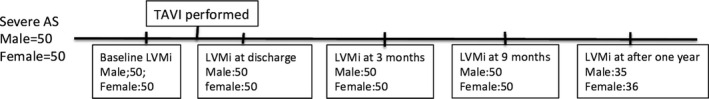
Study flow of LVMi change after TAVI for severe AS patients

A total of 100 severe, symptomatic AS patients (n = 50 men and 50 women) received echocardiograms measured at baseline, discharge, and three and nine months post‐TAVR were enrolled in this study. Echocardiograms were not obtained post‐TAVR for 29 patients after one year for the following reasons: noncardiac‐related deaths (n = 13); cardiac‐related deaths (n = 8); underwent follow‐up at a referral hospital (n = 7) and poor image quality (n = 2). The study flow and baseline characteristics are listed in Table 1 and Figure 1.

**Table 1:** Baseline characteristics of study population

	Male (n = 50)	Female (n = 50)	*P*
Age, y	80.96 ± 8.79	79.46 ± 9.95	.371
Height, mm Hg	164.5 ± 6.2	151.3 ± 4.5	<.001
Weight, kg	65.3 ± 11.6	56.0 ± 10.5	<.001
BMI, kg/m^2^	24.0 ± 3.5	24.62 ± 4.1	.431
Hypertension, n (%)	38 (76)	34 (68)	.504
BSA	1.72 ± 0.17	1.53 ± 0.17	<.001
DM, n (%)	19 (38)	17 (34)	.835
CABG, n (%)	1 (2)	3 (6)	.307
PCI, n (%)	19 (38)	11 (22)	.081
PVD, n (%)	12 (24)	7 (14)	.202
Hyperlipidemia, n (%)	22 (44)	22 (44)	1.000
Euroscore	16.48 ± 12.65	20.94 ± 18.35	.004
CHF, III‐IV	29 (58)	30 (60)	.839
Bicuspid, n (%)	6 (12)	6 (12)	1.000
PLF‐LG AOST, n (%)	16 (32)	17 (34.7)	.872
AF, n (%)	7 (14)	13 (26)	.211
Stroke/TIA, n (%)	13 (26)	7 (14)	.084
Hyperlipidemia, n (%)	22 (44)	22 (44)	1.000
Pulmonary disease	15 (30)	14 (28)	.826
Prior BAV	2 (4)	5 (10)	.436
Prior MI	2 (4)	3 (6)	1.000
Prior CABG, n (%)	1 (2)	3 (6)	.307
Prior PCI, n (%)	19 (38)	11 (22)	.081
Prior BAV	2 (4)	5 (10)	.436
Prior AF	7 (14)	13 (26)	.211
Valve type			
Edward	10 (20)	6 (12)	<.001
23 mm	3 (6)	5 (10)	
26 mm	6 (12)	1 (2)	
29 mm	1 (2)	0	
Metronic	40 (80)	44 (88)	
26 mm	11 (22)	28 (56)	
29 mm	22 (44)	15 (30)	
31 mm	7 (14)	1 (2)	
Vascular access			
Transfemoral, n (%)	42 (84)	45 (89)	.631
Transapical, n (%)	1 (2)	1 (2)	
Direct aortic, n (%)	7 (14)	4 (9)	

Note

Values data are n (%) or mean ± SD.

Abbreviations: AF, atrial fibrillation; AVA, aortic valve area; BAV, balloon aortic valvuloplasty; BMI, body mass index; CABG, coronary artery bypass graft; CAD, coronary artery disease; CHF, congestive heart failure; DM, diabetes; LAD, left atrium diameter; LVEF, left ventricular ejection fraction; LVMi, left ventricle mass index; MPG, mean pressure gradient; PCI, percutaneous coronary intervention; PLF‐LG AOST, paradoxical low‐flow low gradient aortic stenosis; PVD, peripheral vascular disease.

The study population had a mean age of 80.21 ± 8.37 years and an average aortic valve area of 0.70 ± 0.23 cm^2^. The female subjects had higher EuroSCOREs (20.94 ± 18.35 vs 16.48 ± 12.65, *P* = .004) and shorter statures (151.3 ± 4.5 vs 164.5 ± 6.2 cm) and weighed less (56 ± 10.5 vs 65.3 ± 11.6 kg) than the men, though both groups had similar body mass indexes (BMIs). The prevalence of co‐morbidities including hypertension, diabetes, bicuspid aortic valve, history of CAD, previous coronary intervention, peripheral vascular disorder, atrial fibrillation, previous stroke and percentage of stage III‐VI congestive heart failure (CHF) were comparable for both sexes. Although there is a slightly higher percentage of low‐flow low gradient aortic stenosis (PLF‐LG AOST) in woman, their difference is insignificant (34.7% vs 32%, *P* = .872). Echocardiographic analysis revealed narrower aortic roots (30.2 ± 3.5 mm vs 33.3 ± 3.4 mm, *P* < .001) and smaller aortic valve areas in women (0.64 ± 0.21 cm^2^ vs 0.76 ± 0.23 mm^2^, *P* = .007), but indexed aortic valve area, mean pressure gradient, peak pressure gradient, ejection fraction, LV mass and indexed LV mass were similar in both sexes (Table 2). In addition, the valvulo‐arterial impedance (Zva)^15^, ^16^ was estimated and female patients have significant higher (Zva) at baseline before TAVI procedure than male patients (6.4 ± 2.1 vs 5.6 ± 1.7 mm Hg/mL/m^2^, *P* = .039).

**Table 2:** Serial change of parameters of echocardiography after TAVI

	Male, N = 50	Female, N = 50	*P*
Baseline			
Aortic root, mm	33.3 ± 3.4	30.2 ± 3.5	<.001
LAD, mm	43.8 ± 7.6	45.36 ± 9.5	.378
AVA, cm^2^	0.76 ± 0.23	0.64 ± 0.21	.007
AVA/BSA	0.45 ± 0.14	0.41 ± 0.14	.29
MPG, mm Hg	43.0 ± 17.6	49.1 ± 22.5	.136
PPG, mm Hg	72.5 ± 28.8	79.7 ± 35.1	.268
Septum, mm	12.5 ± 1.7	12.4 ± 2.0	.745
PW, mm	12.4 ± 1.7	12.2 ± 1.8	.415
LVEDV, mL	89.6 ± 29.5	67.7 ± 29.2	.001
LVESV, mL	43.3 ± 24.0	32.74 ± 23.5	.037
LVIDd, mm	49.2 ± 7.9	47.3 ± 7.7	.56
LVIDs, mm	31.1 ± 8.8	30.0 ± 9.1	.22
LVEF, %	54.7 ± 11.5	55.2 ± 10.9	.823
LV mass	245.8 ± 75.7	227.3 ± 76.5	.227
LVMi	143.1 ± 39.6	148.3 ± 48.0	.552
Zva, mm Hg/mL/m^2^	5.6 ± 1.7	6.4 ± 2.1	.039
E/E′	22.1 ± 9.6	26.2 ± 11.3	.065
LA volume, mL	76.4 ± 44.9	74.5 ± 54.9	.847
PA pressure, mm Hg	42.4 ± 15.2	48.2 ± 16.8	.098
Discharge			
Aortic root, mm	30.3 ± 4.6	27.1 ± 4.4	.001
LAD, mm	44.8 ± 8.4	45.1 ± 9.6	.858
AVA, cm^2^	1.57 ± 0.39	1.51 ± 0.40	.580
MPG, mm Hg	10.1 ± 4.3	9.5 ± 4.9	.502
PPG, mm Hg	18.6 ± 8.2	17.6 ± 8.6	.572
Septum, mm	12.2 ± 1.9	11.5 ± 1.9	.09
PW, mm	12.2 ± 1.8	11.2 ± 2.0	.013
LVEDV, mL	85.1 ± 32.4	68.2 ± 28.2	.009
LVESV, mL	39.6 ± 20.5	31.6 ± 22.4	.080
LVIDd, mm	49.1 ± 6.9	46.8 ± 7.7	.12
LVIDs, mm	31.6 ± 7.0	28.2 ± 8.8	.035
LVEF, %	55.1 ± 8.8	56.9 ± 9.6	.338
LV mass	237.3 ± 69.3	199.7 ± 68.7	.008
LVMi	138.4 ± 36.9	130.5 ± 43.7	.335
Zva, mm Hg/mL/m^2^	4.5 ± 2.1	4.8 ± 1.7	.434
E/E′	18.6 ± 7.3	24.1 ± 8.6	.003
LA volume, mL	72.6 ± 43.5	70.4 ± 42.9	.799
PA pressure, mm Hg	35.9 ± 9.7	43.7 ± 15.2	.005
2nd follow‐up			
Aortic root, mm	30.0 ± 4.2	27.9 ± 4.7	.016
LAD, mm	42.9 ± 7.5	45.5 ± 9.1	.127
AVA, cm^2^	1.9 ± 0.8	1.6 ± 0.4	.148
MPG, mm Hg	10.1 ± 4.7	9.7 ± 4.9	.691
PPG, mm Hg	18.5 ± 8.9	18.1 ± 9.2	.828
Septum, mm	12.0 ± 2.5	11.1 ± 2.1	.003
PW, mm	11.5 ± 1.8	10.9 ± 1.5	.057
LVEDV, mL	81.3 ± 28.3	67.4 ± 30.8	.027
LVESV, mL	37.7 ± 17.0	31.4 ± 24.0	.152
LVIDd, mm	49.9 ± 6.8	45.5 ± 7.6	.09
LVIDs, mm	31.5 ± 6.7	29.0 ± 8.1	.003
LVEF, %	55.1 ± 7.4	56.3 ± 10.0	.498
LV mass	231.4 ± 72.3	182.8 ± 69.5	.001
LVMi	133.9 ± 37.9	119.1 ± 42.9	.062
Zva, mm Hg/mL/m^2^	4.0.7 ± 1.9	4.2 ± 1.5	.147
E/E′	19.9 ± 8.8	25.3 ± 10.9	.016
LA volume, mL	68.9 ± 37.3	68.4 ± 52.1	.956
PA pressure, mm Hg	36.2 ± 10.1	44.3 ± 16.6	.005
3rd follow‐up			
Aortic root, mm	30.8 ± 4.9	27.2 ± 4.7	<.001
LAD, mm	42.7 ± 9.0	45.5 ± 9.1	.125
AVA, cm^2^	1.7 ± 0.6	1.7 ± 0.6	.629
MPG, mm Hg	9.5 ± 4.6	9.4 ± 4.8	.845
PPG, mm Hg	17.8 ± 7.9	17.5 ± 8.8	.873
Septum, mm	11.7 ± 2.2	10.8 ± 1.9	.021
PW, mm	11.2 ± 1.7	10.7 ± 1.5	.172
LVEDV, mL	83.9 ± 33.0	64.0 ± 32.0	.005
LVESV, mL	38.1 ± 17.9	28.9 ± 23.2	.039
LVIDd, mm	49.0 ± 7.3	45.7 ± 6.6	.03
LVIDs, mm	31.3 ± 7.0	28.2 ± 7.2	.02
LVEF, %	55.5 ± 8.3	57.8 ± 9.2	.211
LV mass	218.0 ± 70.0	178.4 ± 51.5	.002
LVMi	126.7 ± 36.1	116.8 ± 33.5	.171
Zva, mm Hg/mL/m^2^	4.7 ± 1.5	4.3 ± 1.2	.144
E/E′	20.1 ± 11.1	25.2 ± 14.2	.07
LA volume, mL	62.2 ± 39.2	66.1 ± 57.4	.692
PA pressure, mm Hg	35.4 ± 10.1	42.2 ± 15.8	.017
4th follow‐up			
Aortic root, mm	31.6 ± 4.6	26.5 ± 3.8	<.001
LAD, mm	42.0 ± 6.2	47.4 ± 11.6	.018
AVA, cm^2^	1.59 ± 0.48	1.58 ± 0.44	.902
MPG, mm Hg	10.1 ± 6.1	10.1 ± 5.0	.973
PPG, mm Hg	19.0 ± 10.9	18.5 ± 9.3	.844
Septum, mm	11.4 ± 1.8	11.2 ± 2.1	.649
PW, mm	11.5 ± 1.5	10.7 ± 1.4	.207
LVEDV, mL	83.6 ± 34.4	62.6 ± 29.1	.008
LVESV, mL	38.2 ± 22.5	28.7 ± 23.2	.090
LVIDd, mm	48.8 ± 8.1	44.4 ± 7.8	.024
LVIDs, mm	31.2 ± 8.0	28.4 ± 8.3	.17
LVEF, %	56.4 ± 9.1	56.9 ± 9.3	.839
LV mass	210.3 ± 66.7	174.4 ± 47.9	.012
LVMi	121.0 ± 32.1	115.7 ± 30.8	.487
Zva, mm Hg/mL/m^2^	4.5 ± 1.9	4.2 ± 1.8	.497
E/E′	21.1 ± 9.3	23.6 ± 10.5	.326
LA volume, mL	48.9 ± 39.2	54.0 ± 49.1	.626
PA pressure, mm Hg	36.4 ± 9.2	43.5 ± 15.6	.025

Note

Values data are mean ± SD.

Abbreviations: AVA, aortic valve area; LAD, left atrium diameter; LVEF, left ventricular ejection fraction; LVMi, left ventricle mass index; MPG, mean pressure gradient; PW, posterior wall; Zva, valvulo‐arterial impedance.

The procedural details are listed in Table 1. The Edwards SAPIEN valve or SAPIEN XT valve was implanted into 16 patients and the Medtronic CoreValve into 84 patients. In accordance with their narrower aortic annuli, significantly smaller valves were implanted into women. The transfemoral approach was mostly used with 87 patients. The VARC‐2–defined combined safety endpoint at 30 days was comparable for both sexes (Table 3). Moderate paravalvular aortic regurgitation (PVL) occurred in two female patients (4%) and none of the male patients. Of the entire patient population, 5 (10%) required permanent pacemaker implants and 16 (32%) developed new‐onset left bundle branch blocks (LBBB) after the procedure without significant sex‐specific differences.

**Table 3:** VARC‐2 outcomes at 30 d

	Male, N = 50	Female, N = 50	*P*
ARC‐2 defined outcomes at 30 d after TAVI			
CV death, n (%)	0 (0)	0 (0)	ns
Ischaemic stroke, n (%)	1 (2)	0 (0)	ns
Major bleeding, n (%)	3 (6)	1 (2)	.31
Acute kidney injury, n (%)	3 (6)	2 (4)	.65
Coronary obstruction, n (%)	1 (2)	1 (2)	1.0
Myocardial infarction, n (%)	2 (4)	1 (2)	.56
Major vascular complication, n (%)	2 (4)	1 (2)	.56
New permanent pacemaker implantation, n (%)	3 (6)	2 (4)	.64
Valve‐related dysfunction, n (%)	0 (0)	0 (0)	ns
Post‐procedure moderate‐to‐severe PVL, n (%)	0 (0)	2 (4)	ns
New‐onset LBBB, n (%)	7 (14)	9 (18)	.58

Abbreviations: CV, cardiovascular; LBBB, left bundle branch block; PVL, paravalvular leak.

**Figure 2:**
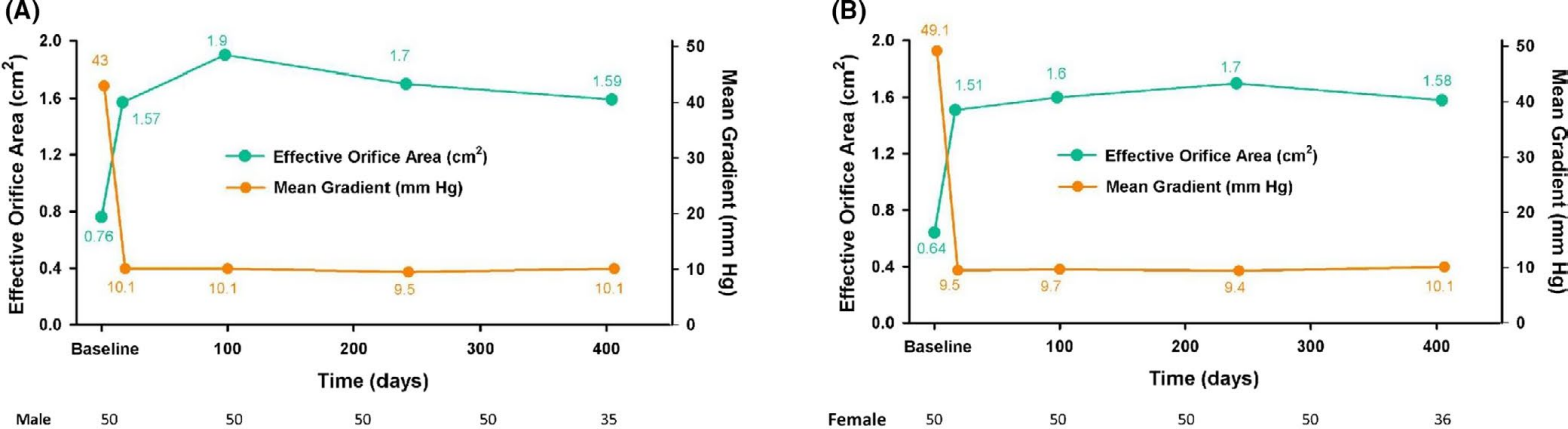
Serial follow‐up of artic aortic valve area (AVA) and mean pressure gradient (MPG) in man (A) and woman (B) after TAVI

**Figure 3:**
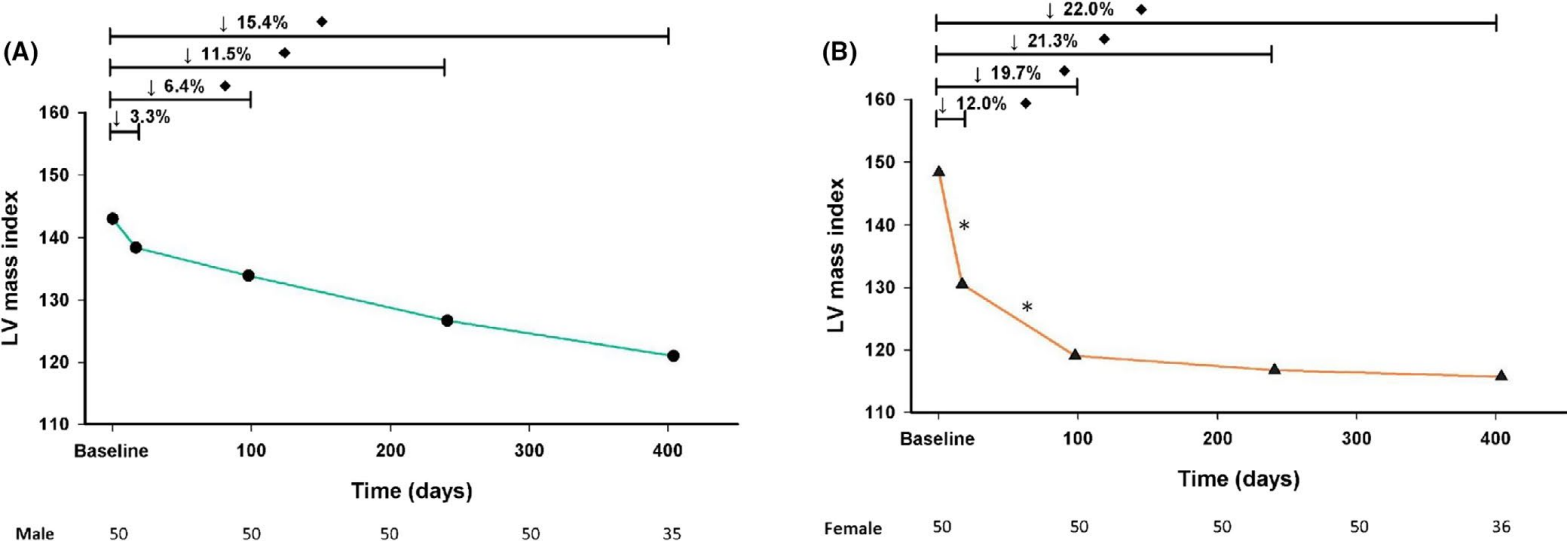
Serial LVMi change after TAVI in man (A) and woman (B). *P < .05 between two LVMi measurements; ^♦^ *P* < .05 compared with baseline LVMi

Table 2 shows the sequential change in the echocardiographic parameters of the LV remodelling after the TAVR procedure. Echocardiography was initially performed just prior to the TAVR procedure, the second time echocardiography at discharge performed at a mean of 16.7 days after the TAVR, the third echocardiography at a mean of 98 days and the fourth a mean of 240.9 days after the procedure (Figure 1). Predictably, the parameters describing AS severity changed profoundly after the TAVR procedure, with a statistically significant increase in AVA and a significant decrease in the transvalvular gradients, which remained consistently stable across the follow‐up period for both sexes (Figure 2A,B). In addition to changes of AVA and pressure gradients, significant reductions in Zva were observed after TAVI as well as left atrium (LA) volume and pulmonary artery (PA) pressure, and Zva remained consistent during the follow‐up period. As shown in Figure 3, the female patients exhibited an early pronounced regression of the LV mass after TAVR and their LVMi decreased from 148.3 ± 48.0 to 130.5 ± 43.7 g/m^2^ (mean change, −17.8 g/m^2^) at only a median of 16.7 days after the procedure (*P* < .001). Significant LVMi regression was observed at the time of discharge in the female patients: the mean fractional LVMi regressed by 12.0% at 16.7 days after TAVR and achieved 22.0% after one year (Figure 3). In contrast, the regression of the LVMi from baseline in men was seen at three months post‐TAVR, decreasing from 143.1 ± 39.6 to 133.9 ± 37.9 g/m^2^ (mean change, −9.2 g/m^2^). The mean fractional decrease of the LVMi was 11.5% at the nine‐month follow‐up and reached 15.4% after one year (Figure 3), suggesting female patients had significantly faster and greater LVMi regression after TAVR (*P* < .05). Table 4 presents the results of the logistic regression analysis, which evaluated the independent predictors of greater early LV mass regression after TAVI. After adjusting for age, sex, BMI, echocardiographic parameters, history of hypertension, diabetes, LV ejection fraction, new‐onset LBBB, moderate‐to‐severe PVL post‐TAVI and the need for permanent pacemaker implantation, only the female sex, LVEF and baseline LVMi were independently associated with greater LVMi regression after TAVR.

**Table 4:** Factors associated with early regression of LV after TAVI within 3 mo

	Model 1			Model 2		
	β	95% CI	*P* value	β	95% CI	*P* value
Age	−.001	−0.006 to 0.005	.801	−.002	−0.008 to 0.003	.388
Female	−.125	−0.213 to −0.037	.006	−.113	−0.203 to −0.023	.025
BMI	.002	−0.01 to 0.014	.72	.005	−0.007 to 0.018	.388
Hypertension	−.001	−0.11 to 0.124	.744	.003	0.097‐0.104	.946
Diabetes	−.01	−0.111 to 0.091	.85	.024	0.075‐0.122	.63
Baseline AVA	.087	−0.114 to 0.288	.393	−.002	−0.246 to 0.242	.987
Mean PPG	−.002	−0.004 to 0	.094	−.001	−0.004 to 0.002	.586
LVEF	−.007	−0.011 to −0.004	<.001	−.007	−0.011 to −0.002	.003
LVMI	−.002	−0.003 to −0.001	<.001	−.003	−0.004 to −0.001	.001
Moderate‐to‐severe PLV	.028	−0.288 to 0.344	.859	.155	−0.135 to 0.444	.29
New‐onset LBBB	−.006	−0.186 to 0.006	.347	−.046	−0.158 to 0.066	.417

Note

Model 1. Crude analysis. Model 2. Adjust with age, female, BMI, history of hypertension, diabetes, baseline AVA, mean PPG, LVEF and LVMI.

## 4 DISCUSSION

Our current result demonstrated that women had favourable reverse remodelling with significant LV mass regression occurring within the first two weeks after the procedure. In contrast, the regression of the LVMi in men seemed to be more gradual. The overall percentage of LVMi regression was greater among women. Additionally, the female sex, better LVEF and baseline LVMi were independently associated with greater early LV remodelling process, indicating that these could be used as indicators for early LV mass regression post‐TAVR in the Asian population.

The pressure overload in the LV caused by AS induces cardiac muscle hypertrophy and interstitial collagen deposition, leading to structural changes in the LV (LV remodelling). LV remodelling alone can greatly impact morbidity in this patient population.^17^, ^18^ TAVR causes an acute decrease in the transvalvular gradient that could lead to unloading the LV and, thus reverses LV remodelling and improves clinical outcomes.^18^

Previous studies have suggested that female sex is associated with better mid‐ and long‐term survival post‐TAVR despite the increased rate of periprocedural complications.^2^, ^3^, ^4^, ^5^, ^6^ There is currently no clear‐cut explanation for why women fare better than men after TAVR. However, the interplay of several risk factors and co‐morbidities, as well as different sex‐related cardiac pathophysiological responses to pressure afterload, are likely involved. Multiple studies have shown that sex‐specific differences exist in terms of the LV response to chronic afterload.^5^, ^19^ Women with severe AS typically manifest more concentric LV geometry, less myocardial fibrosis and better systolic function compared with men.^7^, ^8^, ^9^, ^10^ Surgical studies of patients undergoing AVR have revealed reduced levels of fibrosis in the myocardium biopsied from women and the regression of the myocardial hypertrophy is also more rapid in women.^11^ Stangl et al^20^ showed that, while regression of hypertrophy occurred in both men and women post‐TAVR, the improvement in the ejection fraction was significant only in female patients, potentially reflecting a lower burden of irreversible myocardial damage prior TAVR. Myocardial fibrosis can be identified and quantified pre‐TAVR by cardiac magnetic resonance (CMR). Mid‐wall late gadolinium enhancement (LGE) is predictive of patient outcome and also of the likely improvement in LV remodelling following AVR or TAVR.^21^, ^22^, ^23^ Treibel et al observed that myocardial fibrosis and extracellular matrix expansion were elevated in male patients with severe AS.^23^

Cellular, molecular and neurohormonal mechanisms underlying the differential response of the sexes have been proposed and include increased interstitial fibrosis, greater activation of pro‐fibrotic and pro‐inflammatory pathways, and the differential expression of the androgen and oestrogen receptors, respectively.^10^, ^11^, ^24^, ^25^ There is a large body of evidence linking abnormal myocardial hypertrophy and fibrosis at the molecular level in male patients with AS to the dysregulation of extracellular matrix turnover. Other studies have indicated that the increase in cardiac fibrosis observed in men with AS is associated with increased TGF‐β1 protein expression and SMAD2 phosphorylation.^11^, ^26^, ^27^

The present study provides additional insight into the timeline of LV mass regression after TAVR. It has been well documents in the literature that most of the mass regression occurs within 30 days post‐TAVI, at rates of 3%‐10%.^28^, ^29^ In our study, men and women showed a similar degree of indexed aortic valve area restriction and transvalvular gradients prior to TAVR. While both sexes exhibited increases in the thicknesses of IVS and PW and in the indexed LVH, only women exhibited signs of reverse remodelling almost immediately after the TAVR procedure, with significant LV mass regression occurring within the first two weeks after the procedure. In contrast, the regression of the LVMi in male patients appeared to be more gradual. The significant regression of the LVMi was only observable starting at three months post‐TAVR. Using echocardiography‐based approaches to explore sexual dimorphisms in cardiac remodelling in patients with AS has not been extensively described in the literature. Lindman et al showed that LVMi regression over the first year post‐TAVR occurred in both men and women (*P* < .001 for both) with similar patterns and incremental regressions; however, the overall percentage of LVMi regression was greater in women (*P* = .004). In contrast, Stangl et al found that the LVM and the LVM index had both decreased significantly at the three‐month post‐TAVR follow‐up without relevant differences between the sexes. Furthermore, although there is no difference in paradoxical low‐flow low gradient aortic stenosis (PLF‐LG AOST) percentage between genders, the female patients have higher baseline valvulo‐arterial impedance (Zva). The valvulo‐arterial impedance (Zva) provides an estimate of the global left ventricle (LV) hemodynamic load that results from the summation of the valvular and vascular loads.^15^, ^16^ Additionally, it incorporates stenosis severity, volume flow rate, body size and systemic vascular resistance. High Zva was observed in severe AS and was associated with poor prognosis. It has been reported TAVI cause acute declines in Zva^15^ and the reduction in Zva after TAVI was shown to persist during a 2‐year follow‐up.^16^ In our current study, female patients have significantly higher (Zva) at baseline before TAVI procedure and the Zva significantly decreased after TAVl and remained consistent during follow‐up period, which is in concordance with previous observations. Greater early change of Zva in female gender may contribute to the LV regression after TAVI procedure and further study is needed to confirm this hypothesis.

### 4.1 Study limitations

While our study did not use a matched‐control group design, the baseline risk factors, severity of the AS, included valve area, and pressure gradient were similar between the sexes. Second, most of the cases in this study used self‐expandable valves, but the percentage of balloon‐expendable valves and self‐expandable valves was nonsignificant difference in both groups. Third, in order to evaluate serial LVMi changes after TAVI, only these patients who received regular echocardiography at least nine months in the hospital were analysed in this study, the possible existence of selection bias cannot be excluded. Furthermore, some useful information such as global longitudinal strain by STE or mitral annulus displacement by MAPSE was not recorded in our current study. Finally, longer follow‐up periods will be useful for determining the long‐term impact of LVMi regression and any associated clinical outcomes of the TAVI procedure.

## 5 CONCLUSIONS

In conclusion, our current results demonstrated that female patients with AS had favourable reverse remodelling with greater and earlier LV mass regression relative to the males one. The favourable LV reverse remodelling observed in women might provide a mechanistic explanation for their survival advantage. Furthermore, better LVEF and higher baseline LVMi were associated with earlier LV remodelling post‐TAVR.

## CONFLICT OF INTEREST

There is no conflict of interest.
